# Microwave-Induced Rapid Shape Change of 4D Printed Vegetable-Based Food

**DOI:** 10.3390/foods12112158

**Published:** 2023-05-26

**Authors:** Xiaohuan Chen, Min Zhang, Tiantian Tang

**Affiliations:** 1 State Key Laboratory of Food Science and Resources, School of Food Science and Technology, Jiangnan University, Wuxi 214122, China; 6200112010@stu.jiangnan.edu.cn (X.C.); 7210112048@stu.jiangnan.edu.cn (T.T.); 2 Jiangsu Province International Joint Laboratory on Fresh Food Smart Processing and Quality Monitoring, Jiangnan University, Wuxi 214122, China; 3 China General Chamber of Commerce Key Laboratory on Fresh Food Processing & Preservation, Jiangnan University, Wuxi 214122, China

**Keywords:** 4D printing, shape change, microwave induction, vegetable-based food, temperature distribution

## Abstract

Microwave heating acts as an environmental stimulus factor to induce rapid shape changes in 4D-printed stereoscopic models over time. The influence of microwave power and model structure on the shape change behavior was explored, and the applicability of the deformed method to other vegetable-based gels was verified. The results described that the G′, G″, η, and proportion of bound water of yam gels increased with the increase in yam powder content, and the yam gel with 40% content had the best printing effect. The IR thermal maps showed the microwaves first gathered in the designed gully region caused the swelling phenomenon, which induced the printed sample to undergo a bird-inspired “spreading of wings” process within 30 s. Increasing the microwave power and microwave heating time were able to increase the bending angles and dehydration rates of the printed samples, thus improving the deformed degree and deformed speed. Different model base thicknesses (4, 6, 8, and 10 mm) also had significant effects on the shape change of the printed structures. The efficiency of the shape changes of 4D-printed structures under microwave induction can be judged by studying the dielectric properties of the materials. In addition, the deformed behaviors of other vegetable gels (pumpkin and spinach) verified the applicability of the 4D deformed method. This study aimed to create 4D-printed food with personalized and rapid shape change behavior, providing a basis for the application scenarios of 4D-printed food.

## 1. Introduction

Three-dimension (3D) printing technology, first proposed in 1984, is a development method derived from rapid prototyping technology, which is a type of additive manufacturing technology [1,2]. In recent years, with the continuous understanding of model structures, people are no longer satisfied with the static shapes of 3D-printed food structures. The demand for changes in the dynamic appearances and structural properties of food has promoted the emergence and development of four-dimensional (4D) food printing technology [3,4]. Four-dimensional printing technology enables three-dimensionally printed food to undergo predictable dynamic changes in appearance and structural properties (shape, color, flavor, etc.) over time when it is stimulated correctly in the appropriate environment (temperature, pH, light, moisture content, electromagnetic radiation, etc.), thus allowing for transformation from 3D to 4D [5,6,7]. Therefore, to smoothly print a 4D model, 3 factors need to be met: a stimulus–response material with specific properties, an environmental stimulus that can trigger the state change of the 3D-printed model, and the time demanded for the change in the state of the model [8,9]. 4D-printed food can enhance the interactions between food and consumers, thereby enriching the eating experiences of consumers.

There are two main strategies for studying the shape change achieved by 4D food printing at present: the use of double-layer materials and the design of special model structures. In materials science, the use of two materials with different expansion and shrinkage rates in response to specific stimuli is one of the most common ways to achieve shape changes of materials [10]. This approach has also been used to produce food products. Wang et al. [11] took advantage of the difference in water absorption capacity between gelatin and ethyl cellulose to design a gelatin/ethyl cellulose double-layer structure that could lead to shape change under the stimulation of hydration induction, and on this basis, invented deformable pasta. Chen et al. [12] made use of the different responses of pumpkin and printing paper to temperature to allow pumpkin products combined with printing paper to change shape. There are many ways to induce shape changes by designing special model structures. For instance, ideal deformable models can be obtained by designing filling structures of 3D printed models. Tao et al. [13] changed the product shape by controlling the depth, direction, and number of grooves in the model to control the changes between the dehydration and expansion rates of the material. He et al. [4] achieved shape change behavior of the models under microwave induction by designing filling rates and filling angles of the printed purple potato gel models. The results described that the filling rates and filling angles of the printed models were related to the deformation directions and degrees. However, these studies mainly focused on the shape transformation from planar structures (two-dimension, 2D) to stereoscopic structures (3D), and the amount of time required for shape change was often very long (a few min to more than ten min). On this basis, Shi et al. [14] used the different microwave heating efficiencies of purple potato puree and oil gel to achieve shape changes of the printed stereoscopic structures. Guo et al. [15] realized the directional shape change from 3D structure to 3D structure by using the swelling behavior of the printed food at hot-spots. However, there are still few studies on the rapidly deformed behavior of 4D-printed food.

Microwave is a kind of electromagnetic wave which has the advantages of selective heating, fast heating speed, and short drying time. Microwave heating has important applications in the food industry and in home cooking [16]. During microwave heating, the microwave can penetrate the inside of the material to produce heat so as to allow for volume heating from the inside to the outside [17]. At present, microwave heating technology has been successfully applied to the environment-induced change process of 4D food printing [4,15]. Chen et al. [18] used a microwave to stimulate the decomposition of baking soda in lotus root powder gel to increase the pH of the gel system, thereby inducing the color change process of the lotus root powder gel containing curcumin. Guo et al. [19] used soybean protein extracts and oats as food inks to achieve the shape change of a 4D-printed structure under microwave stimulation.

Vegetables contain a great number of vitamins, minerals, and cellulose, which the human body requires [20]. The daily intake of the vegetable can help to improve the body’s immunity and prevent chronic diseases [21]. The application of vegetables in food printing has a profound influence on the production of personalized nutritious foods. Yams are a kind of food with high nutritional value. Studies have found that yams have antioxidant, anticancer, hypoglycemic, and lipid-lowering functions [22]. Pumpkin and spinach, as common vegetables, have good nutritional function and printing performance, and can also be used as good raw materials for 4D food printing [12].

In this study, microwave heating acted as the environmental stimulus factor for 4D printing to investigate the rapid shape changes of 3D-printed stereoscopic models. The influences of yam powder content on the material properties and printing properties of the mixed gels were studied. The influences of microwave power and model design structures on the deformed behaviors of 3D printed components were investigated, and pumpkin gel and spinach gel were used for the validation tests of microwave-induced shape change. This study aimed to create 4D-printed food with personalized and rapid shape change behavior, providing a basis for the application scenarios of 4D-printed food.

## 2. Materials and Methods

### 2.1. Materials

Yam powder containing 7.6% water, 73.5% carbohydrate, 4.7% protein, and 3.7% crude fat was obtained from Duomai Food Co., Ltd. (Zaozhuang, China). Pumpkin powder containing 7.8% water, 61.2% carbohydrate, 6.4% protein, and 2.6% crude fat was collected from Baofeng Biotechnology Co., Ltd. (Bozhou, China). Spinach powder containing 8.1% water, 55.6% carbohydrate, 19.5% protein, and 2.7% crude fat was collected from Lvshuai Food Co., Ltd. (Xinghua, China). Low acyl gellan gum (food grade) was obtained from Fufeng Biotechnology Co., Ltd. (Urumqi, China).

### 2.2. Preparation of Printing Materials

Yam gel was obtained by mixing the yam powder with deionized water, and the contents of yam powder were 25%, 30%, 35%, 40%, and 45% (*w*/*w*), respectively. The yam gels were gelatinized in a water bath at 75 ± 0.2 °C for 0.5 h. During gelatinization, the container mouth was wrapped with plastic wrap to avoid water loss. According to preliminary test results, 0.5% (*w*/*w*) gellan gum was added to the gelatinized yam gels and mixed evenly to form smooth pastes without any clumps. The deformation model put forward higher requirements for the printability and post-processing abilities of materials. Adding an appropriate amount of gellan gum can enhance the support and stability of the printed samples in the shape change process. Finally, the yam gels were placed in a normal-temperature environment for 30 min to stabilize them for the next study.

Pumpkin gel and spinach gel were prepared by the same preparation process as yam gel to verify the applicability of the 4D deformed method. The selection of printing ink was mainly based on rheological properties, moisture properties, and printing performance. The formula selection of the yam gel will be described later. By the same formula selection method, the contents of pumpkin powder and spinach powder in the pumpkin gel and spinach gel were determined to be 33% and 36% (*w*/*w*), respectively.

### 2.3. Determination of Rheological Properties

A rotating rheometer (DHR-3, TA Instruments, New Castle, DE, USA) was used to analyze the rheological properties of mixed gels with different yam powder contents, referring to the experimental method provided by Yang et al. [23]. The diameter of the experimental parallel plate was 40 mm, and the measurement gap and temperature were 1000 μm and 25 °C, respectively. Firstly, the storage modulus (G′) and loss modulus (G″) of the printing material were determined at angular frequencies ranging from 1 to 100 rad/s by an oscillation test under constant 0.1% strain (within the linear viscoelastic region). A flow scanning test was then carried out at shear rates ranging from 0.1 to 100 s^−1^ to determine the apparent viscosity (η).

### 2.4. LF-NMR Analysis

A low-field nuclear magnetic resonance (LF-NMR) analyzer (MicroMR20-030V-I, Niumag, Shanghai, China) was applied to analyze the moisture statuses and distributions of the mixed gels with different yam powder contents by referring to the experimental method described by Ezeanaka et al. [24]. Approximately 4.00 g of the material was wrapped using plastic wrap and then placed into a nuclear magnetic tube (Ø30 × 200 mm), which was placed into the instrument for detection. The experimental parameters were set as sampling frequency (SW) = 100 KHz, scanning number (NS) = 4, echo time (TE) = 0.400 ms, echo number (NECH) = 1000, and waiting time (TW) = 2500 ms. After the test, the transverse relaxation time (T_2_) of the printing material was obtained by inverting the test results.

### 2.5. Model Design and 3D Printing Process

**Figure 1:**
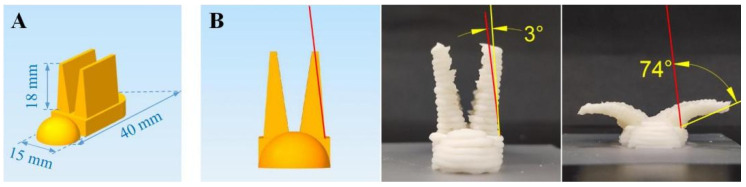
Schematic diagrams of the model’s dimensions (**A**) and the method for determining the bending angles (**B**) of the printed samples.

A cone model (Ø30 mm × 40 mm) was built using Rhinocero 6.0 software (Robert McNeel&Associates, Settle, WA, USA) to examine the printability of the mixed gels with different yam powder contents. A bird model with two wings (40 mm base length, 15 mm base width, and 18 mm wing height) was designed for deformed testing as shown in Figure 1A, with the model base thickness set at 4, 6, 8, and 10 mm, respectively.

The 3D printing process was performed using a printer (Foodbot-Mf, Shiyin, Hangzhou, China) at 25 °C. The printing material was first filled into the printing tube, which was then inserted into the material barrel of the printer for printing. The printer and computer were connected through a USB port, and the printer was controlled by the computer for printing. The model data in STL format were transformed into machine codes using Simplify 3D software, and the X-Y-Z printing paths were automatically generated. Through the preliminary tests, the printing parameters were optimized and set as follows: 1.2 mm layer height and nozzle diameter, 22 mm/s printing speed, and 1.9 mm Z-value.

### 2.6. Microwave Heating and Determination of Temperature Distribution

The printed samples were placed into a microwave oven (M1-L213B, Midea Group Co., Ltd., Foshan, China) with the microwave power set to low fire (70 W), medium fire (350 W), and high fire (700 W). The microwave oven used in this study was a traditional microwave oven, the output power of which was a constant value (700 W). The amount of power was controlled by adjusting the on/off time of microwave emission at different gear positions. According to the preliminary experimental results, the microwave heating times were set as 0, 5, 10, 15, 30, 45, and 60 s, and the samples were immediately taken out for weighing and determination after being heated for the set amount of time.

An infrared thermal imager (IRI-4010, Infrared Integrated Systems Ltd., Northampton, UK) was used to measure the temperature distribution of the microwave-induced samples, referring to the experimental method described by Tang et al. [25]. Only the tested sample was controlled in the measurement background, the focal length was adjusted for measurement, and photos were taken to record the infrared (IR) thermal map. IRISYS 4000 Series Imager software was used to process the map.

### 2.7. Measurement of Bending Angle

Photographs were taken to record the images and shape change behaviors of the samples at different heating times. To quantify the degree of shape change, AutoCAD 2019 software (Autodesk Inc., San Francisco, CA, USA) was purchased to measure the bending angles of 3D-printed samples after microwave heating treatment by referring to the experimental method of Liu et al. [26], as shown in Figure 1B. Both wings of each printed model were measured.

### 2.8. Determination of Dielectric Property

A vector network analyzer (E5062A, Agilent Technologies, Santa Clara, CA, USA) was used to determine the dielectric properties of the printing materials by referring to the experimental method provided by Guo et al. [19]. Firstly, the equipment was calibrated by measuring the air and deionized water. The range of the frequency was set as 2.0 to 3.0 GHz, and the measuring probe contacted the printed samples for testing as far as possible to avoid the existence of air between the measuring probe and the sample, in order to ensure that the dielectric constant (ε′) and loss factor (ε″) reached the accuracy of the measurement.

### 2.9. Statistical Analysis

The experimental data were analyzed using SPSS 25.0 statistical software (IBM, Chicago, IL, USA). Duncan’s test was used for significance analysis at 95% confidence intervals, with *p* < 0.05 indicating obvious differences between the samples. Charts were made using AutoCAD 2019 software and Origin 2018 software (OriginLab Corporation, Northampton, MA, USA). All experiments were performed at least three times in parallel, and the resulting values were represented as mean value ± standard deviation.

## 3. Results and Discussion

### 3.1. Rheological Properties of Printing Materials

**Figure 2:**
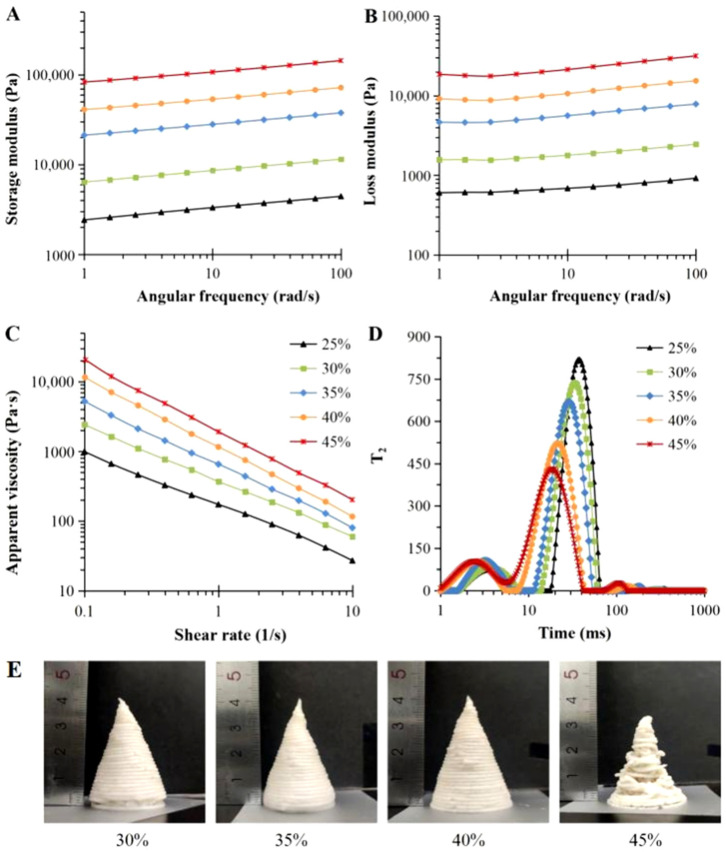
Storage modulus (**A**), loss modulus (**B**), apparent viscosity (**C**), LF-NMR profiles (**D**), and printed results (**E**) of yam gels with different yam powder contents.

The rheological properties of food materials have important effects on the printing performance of 4D-printed samples and the post-processing abilities in the microwave-induced process [27,28]. Figure 2 shows the rheological characteristics of mixed gels with different yam powder contents. The G′ and G″ values of all yam gels presented frequency dependence and content dependence; the modulus values increased as the angular frequency and the yam powder content increased. G′ was related to the mechanical strength of the printed materials and their supporting performance after printing [29,30]. The increase in G′ was conducive to the molding of the printed samples and the maintenance of the structures before inducing the shape change [31,32].

The η and shear-thinning characteristics of the printing materials were obtained to evaluate the extrusion properties of the materials during extruding 3D printing [31]. As shown in Figure 2C, the η values of all printing materials reduced with the addition of the shear rate, showing shear-thinning of the gel, which was propitious to the extrusion of materials from the printing nozzle [28,33]. The shear-thinning property of yam gels was possibly related to the structure and entanglement of polysaccharides’ molecular chains, such as starch and gellan gum in the system and their intermolecular interaction [34]. At lower shear rates, the molecule chains of the polysaccharides were entangled with each other in an irregular manner to create a gel network structure [35]. When the shear rate was increased, the internal network structure and the intermolecular forces were damaged and weakened, which led to decreases in the flow resistance and the η value.

Furthermore, the increase in yam powder content induced an increase in the η value of the material. The elevated yam powder content meant that more starch molecules expanded and split to create a yam puree with higher viscosity in the gelatinization process, and the gel structure of the material was more closely combined, which was also conducive to the supporting ability and printing accuracy of the printed samples after deposition [27]. However, the increase in η put forward higher requirements for the extrusion characteristics of the materials, and the larger the η was, the more difficult the extrusion of the material became, which was also confirmed in the subsequent printing process.

### 3.2. Moisture Characteristics of Printing Materials

LF-NMR spectroscopy revealed the moisture state and moisture distribution of the mixed gels with different yam powder contents (Figure 2D), which correlated with the rheological, mechanical, and printing characteristics of the materials [36]. Different peak areas (A_2_) were obtained to represent the proportions of moisture in different states [37]. Three peaks—T_21_, T_22_, and T_23_—were found in LF-NMR spectroscopy of all of the yam gels, corresponding to the peak areas of A_21_, A_22_, and A_23_, respectively (Table 1). In general, the peak center of T_21_ was located within 1–10 ms, representing the water closely bound with macromolecules in the gel matrix, with low degrees of freedom. T_22_ with a peak center of 10–100 ms indicated semi-bound water, which had a certain fluidity. T_23_ with a peak center of 100–1000 ms represented free water, which was move freely; and this type of water showed easy migration and evaporation [32]. As shown in Figure 2D, with the increase in yam powder content, the peak centers of all T_2_ peaks shifted to the left, while the T_22_ peaks decreased significantly. The freedom of the water in the materials was reduced, and the difficulty of water migration and dehydration evaporation was increased, but the supporting performances of the materials were improved. Table 1 shows the significant effects of yam powder content on the T_2_ and A_2_ values of the mixed gel (*p* < 0.05). T_2_ was related to the freedom and fluidity of the water molecules. The higher the value of T_2_, the higher the freedom and fluidity of the material [25]. The T_21_, T_22_, and T_23_ values of the yam gel were reduced with the increase in yam powder content, meaning that the freedom and fluidity of the water in the material decreased, which was consistent with the left shift result of the T_2_ peak center. As the yam powder content increased, the A_21_ value gradually increased, while the A_22_ value gradually decreased, showing that part of the water changed from semi-bound to bound. This meant that the water, starch, and gellan gum in the system were combined more closely, and the mechanical strength of the material was enhanced along with the increase in yam powder content [35]. These results were identical to the rheological analysis results of the mixed gels, which were conducive to the printing molding and shape maintenance before the induced shape change of the printed models.

### 3.3. Printing Performance of Materials

Four-dimensional food printing technology has put forward higher requirements for the printability and printing precision of materials [38]. To further evaluate the 3D printability of different yam gels, a cone structure was printed for analysis. Figure 2E shows the effects of different yam powder contents on the printing results of the mixed gels. The cone model could be successfully printed with the mixed gels containing 30–40% yam powder content, and the height of the model was close to the designed height of 40 mm. However, with the reduction in yam powder content, the printing accuracy of the yam gel decreased, as did the supporting performance and stability. The model printed by the mixed gel with 30% content of yam powder showed obvious retraction at the bottom, because the reduction of yam powder content reduced the G′, G″, η, and the proportion of bound water in the system, which weakened the mechanical strength and enhanced the fluidity of the gel. Therefore, due to the weak self-supporting performance, the mixed gel with low yam powder content was unable to support its structure after printing, and was prone to collapse to a certain extent. The mixed gel with 25% yam powder content lacked sufficient mechanical strength, making difficult to print. On the contrary, although the mixed gel with 45% yam powder content had high G′ and G″ values, its excessive η also limited the extrusion characteristics of the material. The extrusion of the material was difficult during 3D printing, and there was a serious line fracture phenomenon. The ideal printing material should have the appropriate mechanical strength to improve the printing precision and maintain the stability of the model structure, but at the same time, the η and mechanical strength should not be too high to avoid encountering difficulty during material extrusion [5]. According to the analysis results of the material properties and printing characteristics, the mixed gel with 40% yam powder content was selected as the best printing material in terms of the shape change of 4D-printed food.

### 3.4. Shape Change of the Stereoscopic Model Induced by Microwave

**Figure 3:**
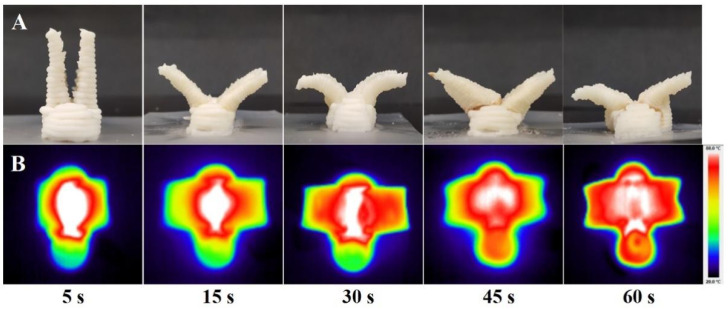
Shape change photographs (**A**) and IR thermal maps (**B**) of the printed samples at 700 W of microwave power (temperature distribution between 20 °C and 80 °C).

Through the active design of the 4D-printed model, a bionic model of a bird with two wings was designed. The connection between the two wings formed a gully structure with a certain angle, which was propitious to the aggregation of microwaves and the formation of hot-spot regions [39]. Microwave heating can make the water molecules inside the food evaporate by heating, resulting in a difference in the environmental pressure between the inside and outside, causing the swelling of the material [40,41]. The gully structure effectively aggregated microwaves and generated a local high temperature in this region, as a hot-spot. The microwave induced the swelling phenomenon of the hot-spot region, which pushed the wings of the bird model to bend outwards and achieve the ideal deformation effect of “spreading of wings” [15]. Figure 3A shows the shape change behaviors of the printed samples at 700 W of microwave power. After microwave heating treatment, the shape of the sample changed and the wings of the model opened outwards. With the extension of the microwave heating time, the shape change degree of the printed structure increased. The two wings of the bird model opened outwards and bent, lowering in height and exhibiting the expected “spreading of wings” behavior. However, when the microwave heating time exceeded 45 s, yellow lines began to appear at the gully part of the printed sample, showing unexpected results. This was caused by the non-uniformity of microwave heating [42].

The IR thermal maps of the printed samples at 700 W of microwave power are shown in Figure 3B. Because of the uneven distribution of the microwaves, they first concentrated in the gully region between the wings of the designed model. The heat of the gully part was greater than that of the other parts, inducing local swelling and pushing the wings to spread out [42]. This result agreed with the expected model design and hot-spot setting. Guo et al. [15] achieved the shape change effect of “flowering” by using the printing method of local swelling induced through non-uniform microwaves. Moreover, with the extension of the microwave heating time, the area range of the hot-spot rose in temperature, inducing a more obvious swelling phenomenon and resulting in a greater shape change. However, when the microwave heating time reached 45 s, the hot-spot area spread to the head area of the bird model, which caused the head gully region to overheat and led to the appearance of yellow lines.

### 3.5. Effect of Microwave Power on Shape Change

**Figure 4:**
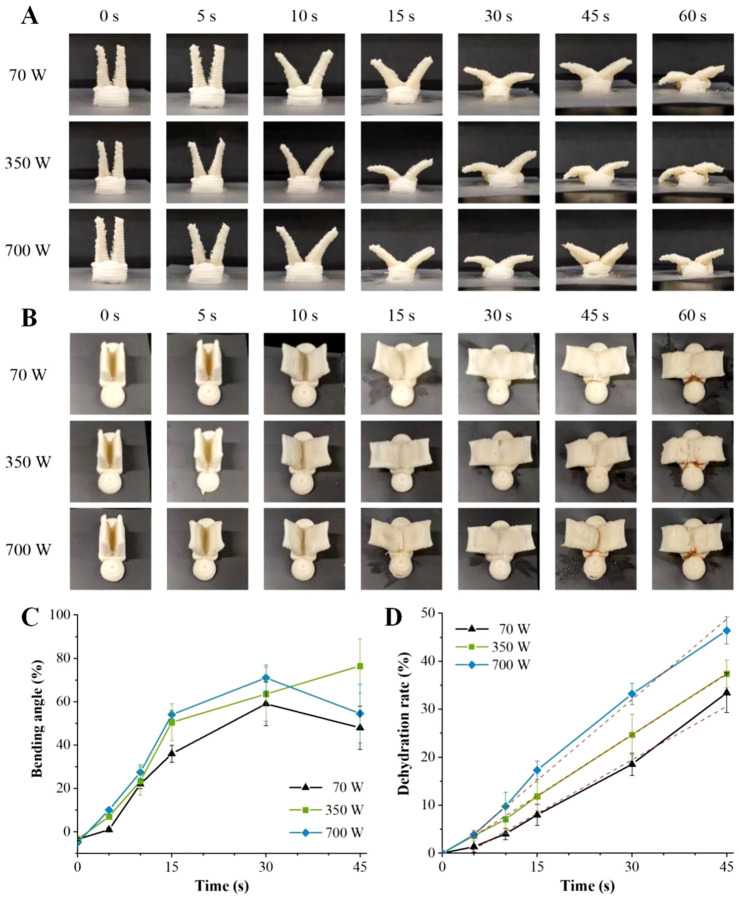
Shape change photographs in front view (**A**) and lateral view (**B**) of the printed samples, and the changes in the bending angle (**C**) and dehydration rate (**D**) at different microwave power levels.

Figure 4 shows the influence of microwave power on the deformation behaviors of the printed structures, which were deformed at different angles under different microwave power levels and microwave heating times. To quantify the shape change behavior, the bending angle was used to represent the degree of shape change (Figure 4C). Due to the ink-swelling effect of food materials, the diameters of the material lines deposited in the printed samples were greater than those in the design models, making the initial bending angles of the printed structures negative [43]. During 0–30 s of microwave heating time, the bending angles of the samples increased as the microwave power and heating time increased, demonstrating power dependence and time dependence. When the heating time reached 30 s and the microwave power was 70, 350, or 700 W, the bending angles of the model wings reached 59.14°, 63.56°, and 71.64°, respectively. When the microwave heating time reached 45 s, the printed models demonstrated unexpected and uncontrollable deformation behavior. This was due to the large amount of materials deposited in the bottom of the model, which concentrated the microwaves and swelled during the microwave treatment. When the microwave heating time was 45 s or 60 s, excessive swelling occurred in the bottom of the models and affected the bending change of the upper wings, as shown in Figure 4A.

The microwave heating process is also a drying and dehydration process [4]. Figure 4D shows the influences of microwave power and microwave heating time on the dehydration rates of the printed food. The dehydration rates of the printed food raised with the increase in microwave heating time, in a time-dependent manner. The linear fitting of the dehydration rate–microwave heating time curves at 70, 350, and 700 W of microwave power showed strong correlations with the fitting values (R^2^) of 0.994, 0.999, and 0.989, respectively. Microwave heating mainly caused high-frequency reciprocating motion or oscillation of the polar substances in the materials through the electromagnetic field, as well as the rotation of dipoles (such as water molecules). The polar molecules or dipoles then showed mutual friction or collision, thus consuming electric energy, transforming into heat energy, and causing water to evaporate [16,44]. With the extension of heating time, water molecules absorbed more microwave energy, which caused increases in friction heat and temperature, thus increasing the dehydration rates. Microwave power also showed an obvious influence on the dehydration rates of the printed food (*p* < 0.05), and the dehydration rates of the printed samples were raised with the increase in microwave power. With 45 s of microwave heating time, the maximum dehydration rates of the printed samples at 70, 350, and 700 W of microwave power were 33.44%, 37.41%, and 46.40%, respectively.

### 3.6. Effect of Model Structure on Shape Change

**Figure 5:**
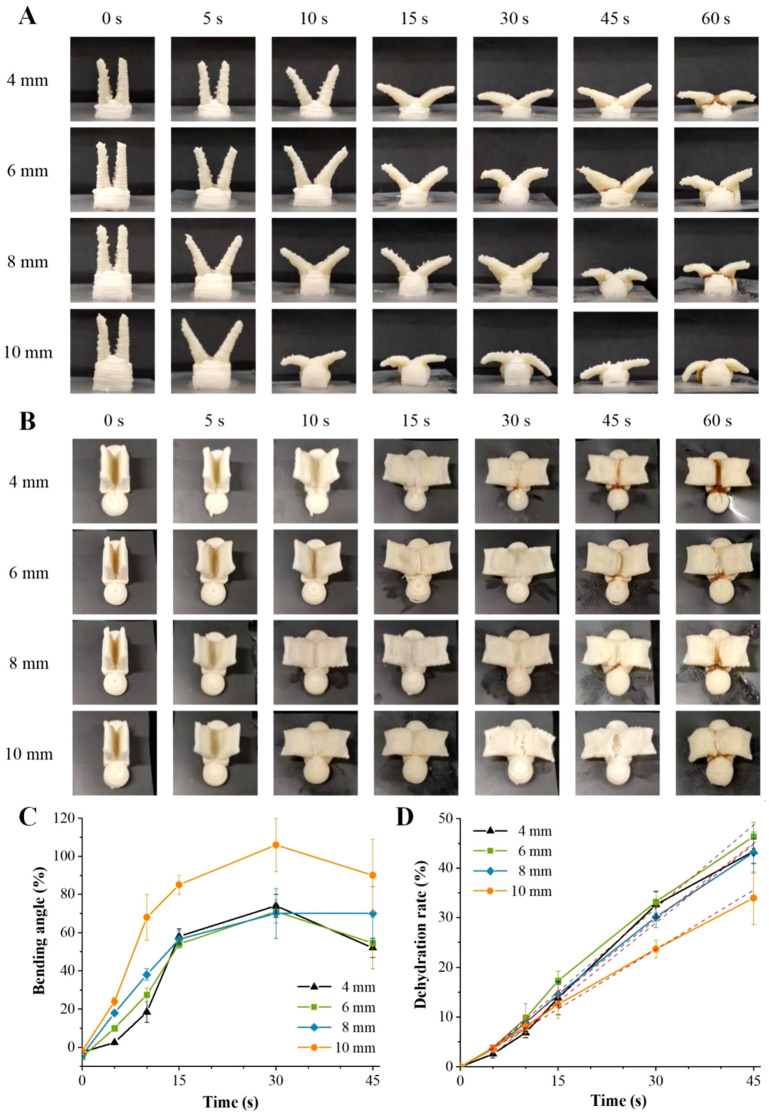
Shape change photographs in front view (**A**) and lateral view (**B**) of the printed samples, and the changes in bending angle (**C**) and dehydration rate (**D**) with different model structures.

The pictures depicting the shape changes of the printed structures with different model base thicknesses under 700 W of microwave power are shown in Figure 5A–C, which display the influence of the model base thickness on the deformed angle of the printed sample. During 0–30 s of microwave heating time, the bending angles of the printed structures showed an increasing trend with time evolution. Within 0–15 s, the bending angles of the printed structures rose as the model base thickness rose. The increase in the base thickness provided the model base with a larger volume, which then absorbed more microwave energy to induce a greater swelling phenomenon during microwave heating and to further increase the degree of shape change [15].

However, when the microwave heating time was 15 s or 30 s, there was no distinct difference in the deformed angles of the printed samples with base thicknesses of 4, 6, and 8 mm (*p* > 0.05), while the bending angle of the sample with a base thickness of 10 mm was significantly larger than that of other samples, showing a greater deformed degree. As shown in Figure 5B, when the microwave heating time reached 30 s or 45 s, the material deposited between the two wings part of the printed sample with a thickness of 10 mm showed visible fractures, which was related to the overheating temperature in the gully region. At this time, the bending angle of the wing part of the printed sample without traction from the model base was not only affected by the driving force produced by the swelling of the gully part, but also caused to collapse downward by the gravity effect of the wing part itself, showing a greater bending angle. Moreover, the bending angles of the printed structures with different base thicknesses increased or decreased at 45 s, at which time the bending angle of the printed structure was positively correlated with the base thickness. The maximum angles of the models with 4, 6, 8, and 10 mm of base thickness were 74.85°, 71.64°, 70.58°, and 106.42° within 45 s of microwave heating time. As the base thickness was raised from 4 mm to 10 mm, the deformed angle increased by 1.42 times.

Figure 5D shows the influence of model base thickness on the dehydration rate of the printed samples. The dehydration rates of the printed food were positively correlated with microwave heating time, and the R^2^ of the dehydration rate–microwave heating time curves of the samples with thicknesses of 4, 6, 8, and 10 mm were 0.991, 0.994, 0.998, and 0.999, respectively. The base thicknesses (6, 8, and 10 mm) also had a certain influence on the dehydration rates of the printed food samples, which reduced with the increase in the base thickness. This was because microwave heating was volumetric, and the microwaves directly entered the interiors of the substances and interacted with each other. The temperature gradients of the material subjected to microwave were high on the inside and low on the outside [17]. The volume of the printed model with a larger base thickness was greater, and the discharge rate of the internal water vapor was slowed down by the material. The exception was that in the early stage of microwave heating (0–10 s), the printed sample with a thickness of 4 mm showed a low dehydration rate due to its weak microwave absorption ability. After 10 s, the dehydration rate of the printed sample was accelerated, and yellow lines appeared at 30 s, which was the earliest compared with the other samples (Figure 5).

### 3.7. Shape Change Behavior of Other Vegetable Gels

**Figure 6:**
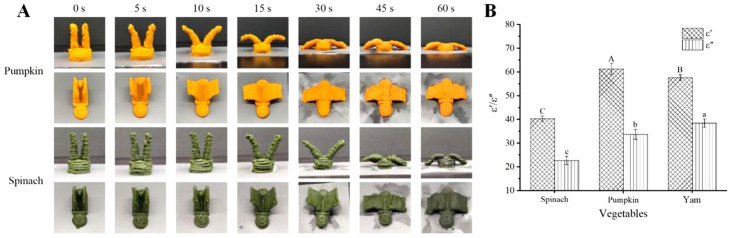
Shape change photographs (**A**) and dielectric properties (**B**) of different vegetable materials. Different letters indicate significant differences between values (*p* < 0.05).

Figure 6A shows the shape change behaviors of the printed samples prepared with pumpkin gel and spinach gel at 700 W of microwave power. Under the action of the microwave, the wings of the bird models opened outwards along with time evolution and the “spreading of wings” behavior of the bird model was simulated, which verified the feasibility and applicability of the microwave-induced shape change method of the 4D stereoscopic model. The bending angle of the printed model rose over time. Compared with the deformed behavior of the sample printed with yam gel, shown in Figure 3, the magnitude of the deformed efficiency of different vegetable gels was pumpkin gel > yam gel > spinach gel. The times to reach the maximum controllable and ideal bending angles of the samples printed with pumpkin gel, yam gel, and spinach gel were 15 s, 30 s, and 30 s, respectively, and the corresponding maximum bending angles were 76.17°, 71.64°, and 41.47°, respectively. This was related to the dielectric properties of the vegetable gels.

In the process of microwave heating, the difference in materials directly affect the microwave heating efficiency, which can be essentially determined by the dielectric property of the food materials. The dielectric property is the response of bound charges in the molecules to the applied electromagnetic field [45]. The dielectric properties ε′ and ε″ determined the interactions between the electromagnetic field and the materials, representing the capacity of the materials to store electromagnetic energy and convert it into thermal energy, respectively [16]. The dielectric properties of the three vegetable gels are shown in Figure 6B. The ε′ values, from high to low, were arranged in the following order: pumpkin gel (61.33) > yam gel (57.67) > spinach gel (40.33). The ε″ values, from high to low, were in the order of yam gel (38.33) > pumpkin gel (33.67) > spinach gel (22.67). The differences in the dielectric properties of the materials were mainly related to the type of materials and their material characteristics (bulk density, water content, etc.) [45]. As a kind of polar molecule, water molecules moved under the action of the microwaves and produced a friction effect, which increased the internal energy of the materials and achieved the effect of rapid heating [14]. The ε″ indicates the capacity of the food material to absorb microwaves, and the greater the ε″ value, the faster the heating rate [35]. Compared with spinach gel, the yam and pumpkin gels exhibited stronger dielectric properties and higher microwave absorption properties, corresponding to higher microwave heating efficiency and 4D change efficiency. The results demonstrated that the deformed efficiency of the 4D-printed samples under microwave induction can be predicted by studying the dielectric properties of the materials.

## 4. Conclusions

The yam powder content affected the printing effect of the mixed gel by influencing its material characteristics. The increase in yam powder content optimized the rheological and moisture characteristics of the materials, but also increased the difficulty of extrusion while improving the printing accuracy. Gel mixed with 40% yam powder content was used as 4D printing ink. A bird model with two wings was designed, and the shape change behavior and IR thermal map of the printed models demonstrated the success of the method, inducing a shape change by forming local hot-spots at the pre-designed gully regions. Increasing the microwave power and heating time increased the bending angles and dehydration rates of the printed samples, and improve the degree and speed of the shape change. However, when the microwave heating time exceeded 45 s, the hot-spot area expanded greatly, causing unexpected yellow lines and affecting the deformed behavior of the printed sample. The model’s base thickness also had an obvious influence on the deformed degrees and dehydration rates of the printed samples. Moreover, the microwave-induced 4D shape change method was also applicable to other vegetable-based gels, and the dielectric properties of the materials affected the microwave heating effect, which could be used to predict the efficiency of the 4D change. In this study, rapid and controllable shape changes of printed food within 30 s were achieved through microwave induction so as to enrich the application scenarios of 4D-printed food.

## Figures and Tables

**Table 1:** LF-NMR analysis of different yam gels.

	T_21_ (ms)	T_22_ (ms)	T_23_ (ms)	A_21_ (%)	A_22_ (%)	A_23_ (%)
25%	7.58 ± 0.56 ^a^	48.30 ± 0.00 ^a^	138.07 ± 21.29 ^a^	10.79 ± 1.63 ^e^	95.90 ± 1.19 ^a^	0.32 ± 0.03 ^d^
30%	8.25 ± 0.66 ^a^	47.46 ± 1.21 ^a^	143.23 ± 14.46 ^a^	12.21 ± 1.16 ^d^	88.97 ± 1.63 ^ab^	0.24 ± 0.03 ^d^
35%	6.91 ± 1.54 ^a^	43.19 ± 0.77 ^b^	102.64± 13.74 ^b^	13.92 ± 1.05 ^c^	87.47 ± 1.17 ^b^	0.95 ± 0.07 ^b^
40%	4.87 ± 0.46 ^b^	35.70 ± 0.71 ^c^	89.93 ± 5.56 ^bc^	16.00 ± 0.21 ^b^	83.05 ± 0.26 ^c^	0.50 ± 0.07 ^c^
45%	4.60 ± 0.03 ^b^	36.43 ± 0.00 ^c^	74.83 ± 9.92 ^c^	19.18 ± 0.08 ^a^	79.19 ± 0.05 ^d^	1.23 ± 0.14 ^a^

Different letters in the same column indicate significant differences between values (*p* < 0.05).

## Data Availability

The data are available from the corresponding author upon suitable request.
